# The Way of the Transgressor Is Hard

**Published:** 1897-09

**Authors:** 


### THE WAY OF THE TRANSGRESSOR IS HARD.

Dr. H. E. Wand, formerly of Philadelphia, recently of Newtown, Pa., must surely have realized ere this "that the way of the transgressor is hard." His recent incarceration in the jail of Chester County, for the forging and raising of checks, the imposition of his worthless checks on his colleagues, and the securing of loans of instruments and not returning them, and many other misdeeds wholly unprofessional, have brought to him, and are sure to bring suffering to those who are so forgetful of self-respect and duty to their colleagues, and it should be a lesson to those who, unmindful at times of the danger, find themselves in perilous positions following a digression from the pathway of professional duty and ethics.

